# Effects of Free and Nanoencapsulated Benznidazole in Acute *Trypanosoma cruzi* Infection: Role of Cholinergic Pathway and Redox Status

**DOI:** 10.3390/ph17101397

**Published:** 2024-10-19

**Authors:** Aniélen D. da Silva, Mateus Fracasso, Nathieli B. Bottari, Taís V. Palma, Ana M. Engelmann, Milagros F. V. Castro, Charles E. Assmann, Vitor Mostardeiro, Karine P. Reichert, Jelson Nauderer, Marcelo L. da Veiga, Maria Izabel U. M. da Rocha, Luiz Claudio Milleti, Gabriella B. das Neves, Samanta Gundel, Aline F. Ourique, Silvia G. Monteiro, Vera M. Morsch, Maria Rosa Chitolina, Aleksandro S. Da Silva

**Affiliations:** 1 Department of Biochemistry and Molecular Biology, Universidade Federal de Santa Maria, Santa Maria 97105-900, RS, Brazil; anielen.dutra@gmail.com (A.D.d.S.); mateus.fracasso1@gmail.com (M.F.); tais.palma58@ufsm.br (T.V.P.); ana.engelmann@ufsm.br (A.M.E.); mifave_11@hotmail.com (M.F.V.C.); charles.ufsm@gmail.com (C.E.A.); vitormostardeiro@gmail.com (V.M.); kakareichert@yahoo.com.br (K.P.R.); jelsonnauderer@gmail.com (J.N.); veramorsch@gmail.com (V.M.M.); mariachitolina@gmail.com (M.R.C.); 2 Department of Microbiology and Parasitology, Universidade Federal de Pelotas, Pelotas 96015-560, Brazil; nathieli_bb@hotmail.com; 3 Department of Pathology, Universidade Federal de Santa Maria, Santa Maria 97105-900, RS, Brazil; marcelo.veiga@ufsm.br (M.L.d.V.); bebelugalde@gmail.com (M.I.U.M.d.R.); 4 Department of Animal Production, Universidade do Estado de Santa Catarina, Lages 88520-000, SC, Brazil; luiz.miletti@udesc.br (L.C.M.); gabriellabassidasneves@gmail.com (G.B.d.N.); 5 Center Science Heath, Universidade Franciscana, Santa Maria 97010-491, RS, Brazil; samantagundel@hotmail.com (S.G.); alineourique@gmail.com (A.F.O.); 6 Department of Microbiology and Parasitology, Universidade Federal de Santa Maria, Santa Maria 97105-900, RS, Brazil; sgmonteiro@uol.com; 7 Department of Animal Science, Universidade do Estado de Santa Catarina, Chapecó 89815-630, SC, Brazil

**Keywords:** nanocapsule, AChE, oxidant, benznidazole, Chagas disease

## Abstract

**Background/Objectives**: The *Trypanosoma cruzi* infection promotes an intense inflammatory process that affects several tissues. The cholinergic system may exert a regulatory immune response and control the inflammatory process. This study aimed to evaluate the comparative effect of free and nanoencapsulated benznidazole in acute *T. cruzi* infection to assess hematological, biochemical, and oxidative status triggered by the cholinergic system. **Methods**: For this, fifty female Swiss mice were distributed in eight groups, i.e., uninfected and infected animals under four treatment protocols: untreated (control—CT); vehicle treatment (Eudragit L 100—EL-100); benznidazole treatment (BNZ); and nanoencapsulated benznidazole treatment (NBNZ). After eight treatment days, the animals were euthanized for sample collection. **Results**: The peak of parasitemia was at day 7 p.i., and the BNZ and NBNZ controlled and reduced the parasite rate but showed no efficacy in terms of total elimination of parasites analyzed by RT-PCR in both infected groups. The infection promotes significant anemia, leukopenia, and thrombocytopenia, which the BNZ improves. There was an increase in AChE activity during infection, leading to a pro-inflammatory response and an increase in M1 and M2 mACh receptors in the BNZ group, showing that the treatment interacted with the cholinergic pathway. In addition, a pro-oxidative response was characterized in the infection and mainly in the infected BNZ and NBNZ groups. The histopathological analysis showed significative splenomegaly and inflammatory infiltrate in the heart, liver, and spleen. **Conclusions**: The administration of the BNZ or NBNZ reverses hematological, hepatic, and renal alterations through cholinergic signaling and stimulates a pro-inflammatory response during acute *T. cruzi* infection.

## 1. Introduction

*Trypanosoma cruzi* is the etiological agent of Chagas disease (CD), transmitted by a vector in America; the infection could occur by maternal–fetal transmission, orally, accidentally, or even by blood transfusion. Naturally, protozoans have tropism for organs such as the heart, brain, intestine, and esophagus, affecting many people worldwide [1]. In Brazil, the highest mortality of people with CD is concentrated mainly in the northern and northeastern states [2]. The protozoan has a complex evolutionary cycle and comprises different stages of development. Among its forms are the infective metacyclic epimastigote and trypomastigote forms in the insect vector and the amastigote and blood-dwelling trypomastigote forms in the vertebrate host [3]. The amastigote form can be found in muscle cells [4] and can remain dormant for a long time without causing damage to the host [4,5]. The trypomastigote form of *T. cruzi* mainly infects macrophages, fibroblasts, and muscle tissues [4]. When invading cells, a lysosomal vacuole will engulf the trypomastigote form. Still, it can leave its acidic environment and produce the amastigote form in the cytoplasm, where it will replicate [6,7]. This process may occur during the S phase of the cell cycle, in which glucose and energy are consumed, triggering the MAPK signaling pathways, which results in cell growth and differentiation [4].

Nifurtimox and benznidazole (BNZ) are the options for treatment in the acute and chronic stages of the disease. Benznidazole is a drug derived from nitroimidazole, with low solubility in water [8] and has good biodistribution (92%) in organs such as the spleen, liver, lungs, kidneys, heart, and brain (organs most affected by *T. cruzi* infection), but has low absorption during first-pass metabolism in the liver [9,10]. Although there is a lower therapeutic response after the chronic phase, studies indicate a minor change in the electrocardiographic pattern and a delay in the onset of clinical complications among patients treated with benznidazole. It is a prodrug activated by type I nitroreductase, expressed by trypanosome, producing a cytotoxic and mutagenic compound [9]. According to research, benznidazole acts through the oxidation of free nucleotides, the incorporation of which in the DNA replication process can lead to breaks in the DNA double strand in *T. cruzi*, which can be potentially lethal [11].

Chagas disease has many obstacles in its treatment, such as the dose used, the scarce study of new drugs, and especially the adverse effects caused by the BNZ, such as hepatotoxicity, skin reactions, and changes in hematological parameters, among others. Therefore, new therapeutic approaches that regard effectiveness against *T. cruzi* are necessary. In this sense, nanotechnology becomes essential for reducing the dose and improving the drugs’ solubility, absorption, and bioavailability. Recent studies have already indicated that nanotechnology approaches have shown crucial results in the search for strategies that can enhance the performance of the BNZ against *T. cruzi* [12], and these studies include efficacy, toxicity, stability, and drug bioavailability. However, the more significant concern remains about drug targeting and resistance mechanisms of the parasite in conjunction with a prolonged release compared to conventional drugs [13,14].

During intracellular infection by the parasite, an innate immune response is triggered, releasing pro-inflammatory cytokines, which may induce enteric, cardiac, and neuronal damage [15]. Once into the intracellular space, *T. cruzi* increases the formation of reactive oxygen species (ROS) by host cells due to the stimulus of inflammatory mediators, such as cytokines and chemokines, which results in oxidative stress [16]. Oxidative stress is considered a host defense mechanism against the parasite in the acute phase of infection, besides contributing to the development of tissue damage [17,18]. In contrast to *T. cruzi* presence, persistence, and inflammatory processes, anti-inflammatory responses limit excessive inflammation, cellular damage, and apoptosis [19].

The anti-inflammatory cholinergic pathway is a regulatory system involved in immune responses in CD, as reported in a previous study by our group [20]. Immune cells have a complete cholinergic system composed of acetylcholine (ACh), choline acetyltransferase (ChAT), nicotinic and muscarinic receptors (nAChR and mAChR), and acetylcholinesterase (AChE) enzyme [21,22]. ACh is a central and peripheral nervous system neurotransmitter [23]. In addition, this molecule is involved in regulating immune functions [21], showing anti-inflammatory properties [24]. On the other hand, the enzyme butyrylcholinesterase (BChE: EC 3.1.1.8) is present in the intestine, liver, kidneys, heart, lungs, and serum [25]. Despite hydrolyzing esters such as butyrylcholine, BChE can act in the degradation of ACh during a situation of inhibition or absence of AChE [26,27]. Studies in the literature have already demonstrated changes in the activity of acetyltransferase and AChE enzymes related to trypanosomiasis [20,28].

Nanostructuring is an increasingly common practice in pharmacology due to its therapeutic benefits [29]. Therefore, this study aimed to evaluate whether the protocol with nanoencapsulated benznidazole enhances curative efficacy in acute *T. cruzi* infection compared to free benznidazole and the effects of therapies on the cholinergic system response, hematological and biochemical parameters, and oxidative status.

## 2. Results

### 2.1. T. cruzi Infection Confirmation

**Figure 1:**
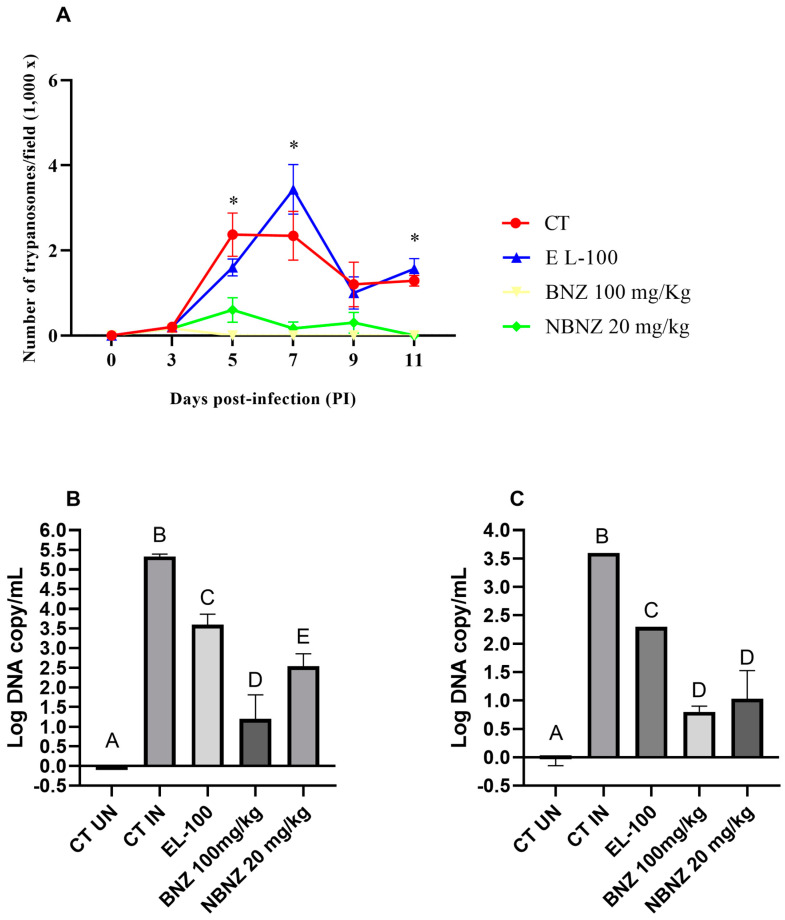
*T. cruzi* infection in mice: (**A**) parasitemia assessment of animals infected by *T. cruzi* (Y strain) exposed to BNZ protocol; (**B**) qPCR of cardiac tissue; and (**C**) qPCR of cortex tissue. Differences were considered statistically significant when * *p* < 0.05 (illustrated by different letters on the bars A–E). Data are presented as mean ± S.E.M.

The parasitemia evolution (Figure 1A) was monitored through a microscope, and the parasitic count was collected in a blood sample by tail vein puncture. The measurement was performed every day until the infection was confirmed; after this, it was executed every two days. The first positive parasitemia analysis was obtained on day 3 p.i., and the peak of parasitemia occurred between days 5 and 7 p.i.; after this period, a possible decrease in parasitemia was observed. The BNZ and NBNZ groups presented a similar standard with low parasitemia at zero on days 5 and 11 p.i., respectively. The BNZ group presented a diminished parasitic load compared to the NBNZ group. There was a significant decrease in parasitemia in the BNZ and NBNZ groups compared to the CT group on days 5, 7, and 11 p.i.; the same was observed compared to the EL-100 group at these time points. Moreover, no differences were observed between the CT and EL-100 groups (*p* > 0.05).

In addition, an RT-PCR was performed to confirm the presence of the parasitic infection, and genomic DNA was quantified in cardiac (Figure 1B) and cortex (Figure 1C) tissues. The quantification of parasitic DNA copies was plotted in a standard curve based on Ct values about the log of DNA copy in the sample. The coefficient of determination (R2) was 0.9919. It was possible to observe a lower number of significative DNA parasite copies in the EL-100, BNZ, and NBNZ groups compared to the infected CT group in both the heart and cortex tissues. All treatments present significant differences in the cardiac tissue, but the lower quantities of parasites occurred in the BNZ followed by the NBNZ. In the cortex sample, the lowest parasite DNA copies were present in the BNZ and NBNZ groups, with no difference observed between them.

### 2.2. T. cruzi Infection Promotes Anemia, Thrombocytopenia, and Granulocytopenia

The erythrogram parameters are shown in Table 1. There was a significant difference between the uninfected and infected groups. The total erythrocytes, hemoglobin, and hematocrit were lower in the infected animals than in the uninfected animals. Furthermore, the BNZ (100 mg/kg) infected group had an elevation of these parameters compared to other infected treatment groups. Still, there was no difference between the uninfected and infected BNZ (100 mg/kg) groups. Furthermore, the number of platelets was lower in the positive control group (the infected group) than in the uninfected group. The same can be observed for platelets in the NBNZ (20 mg/kg) group.

The leukocyte number and its differential count (Table 1) were evaluated in uninfected and infected animals exposed to the BNZ. The CT group shows a lower white cell number tendency in infected animals than in uninfected animals in all treatments. No differences were observed pertaining to the total leukocyte number in the uninfected CT, EL-100, and BNZ groups when compared to the corresponding infected groups. The total leukocyte number was higher in the infected NBNZ group compared to that in the uninfected NBNZ group and the infected CT group.

The granulocyte number was lower in the infected CT, EL-100, groups compared to their corresponding uninfected groups; this parameter also demonstrated significance in the infected NBNZ group compared to the uninfected NBNZ and infected CT groups. The lymphocyte number did not differ significantly between uninfected and infected animals in the CT, EL-100, and BNZ groups. On the other hand, the NBNZ showed a significant lymphocyte number in the infected group compared to the uninfected NBNZ group and the infected CT group. There were no differences in the monocyte number of the infected CT, EL-100, BNZ, and NBNZ groups compared to that of their corresponding uninfected groups.

### 2.3. Hepatic and Renal Alterations Occur Even with Treatment

The biochemical parameters related to hepatic and renal functions were analyzed in the serum sample (Table 2). It was possible to note that the AST and ALT activities of the infected groups were higher compared to those of the uninfected groups in each treatment protocol. The creatinine plasmatic level was higher in the infected CT and BNZ groups than in their corresponding uninfected groups.

The infected CT group showed higher urea serum levels than the uninfected group. The same condition was observed in the uninfected and infected EL-100 groups. However, the NBNZ promoted lower urea levels in the infected group than in the uninfected group. At the same time, all treatment protocols showed lower urea levels in the infected groups compared to the infected CT group.

### 2.4. Benznidazole Improves Cholinergic Signaling

**Figure 2:**
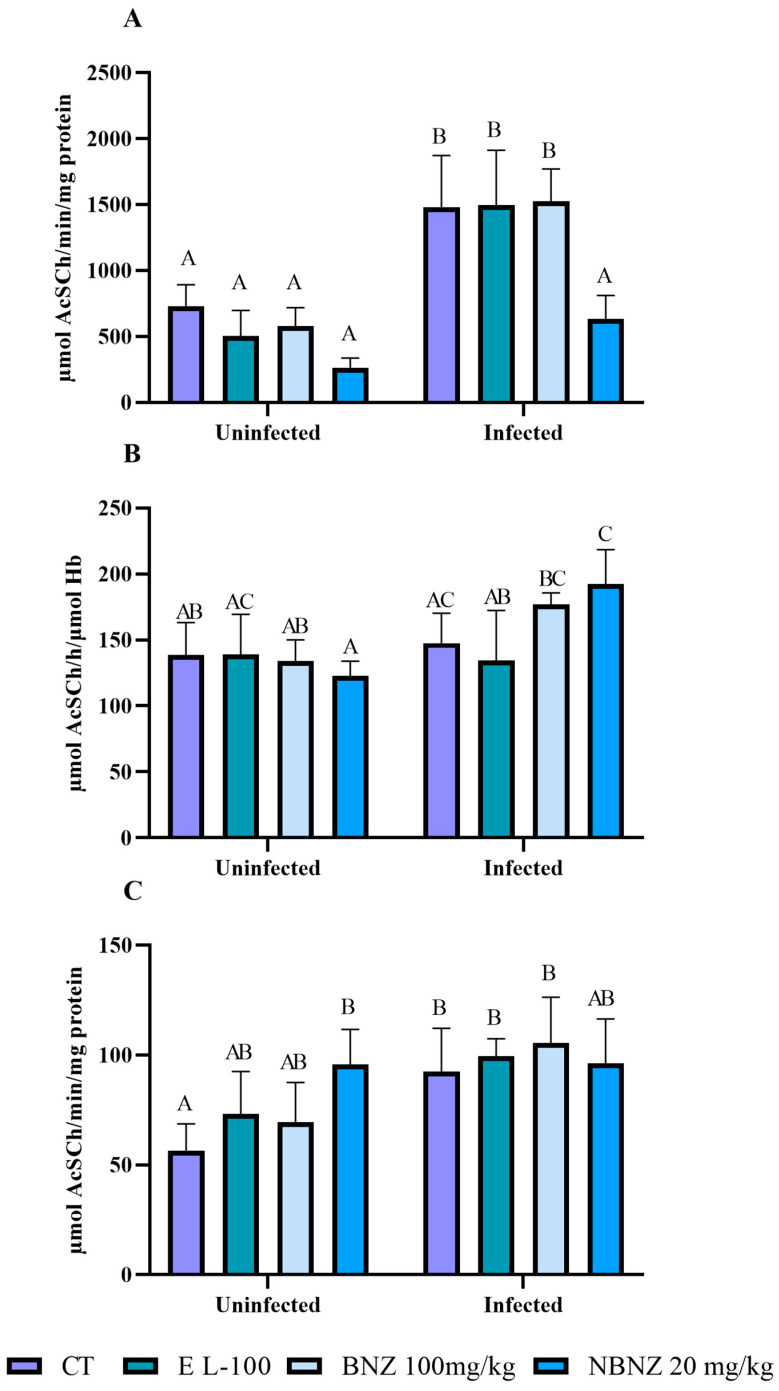
Acetylcholinesterase activity during experimental *T. cruzi* infection in Swiss mice lymphocytes (**A**), erythrocytes (**B**), and cortex (**C**). Bars indicate mean ± SD. The differences are considered statistically significant when *p* < 0.05 and are demonstrated by different letters (A–C); the same letters represent no difference between groups (AB, BC, and AC).

To evaluate the interaction of cholinergic activity in *T. cruzi* infection, the acetylcholinesterase and butyrylcholinesterase activities were assessed in lymphocytes, total blood, and the cortex. The lymphocytes’ AChE enzyme activity was higher in the infected CT group than in the uninfected CT group. The same situation was observed in the comparison between the infected EL-100 and the uninfected EL-100 group and the infected BNZ and the uninfected BNZ group (Figure 2A). However, the NBNZ promotes a lower AChE activity in the infected group than in the infected CT group.

On the other hand, no alterations in the AChE blood activity (Figure 2B) were observed between the infected and uninfected groups of CT, EL-100, and BNZ. However, the infected NBNZ group showed a significant AChE enzyme activity compared to the uninfected NBNZ group. It was observed that there were no significant differences between treatments of infected groups. The enzyme activity in the cortex (Figure 2C) showed elevation only for the infected and uninfected CT groups.

**Figure 3:**
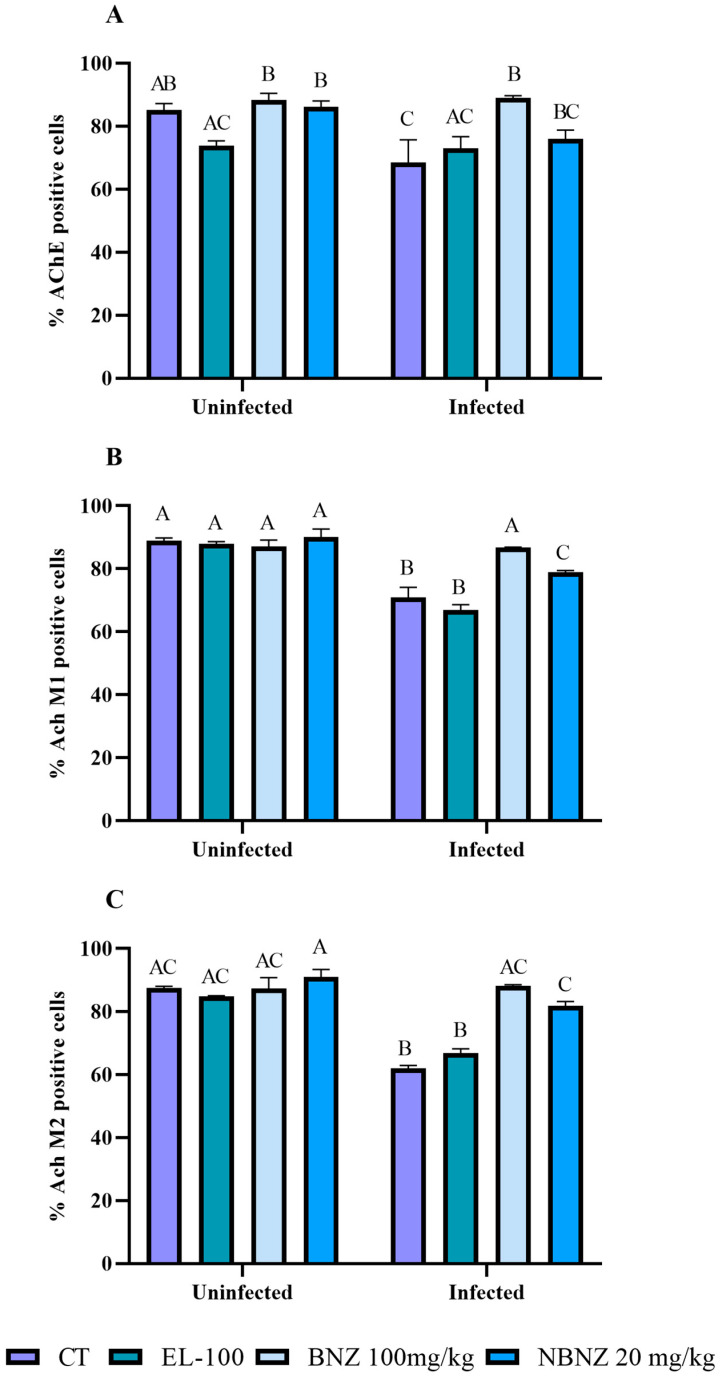
Cytometric analysis of lymphocytes’ cholinergic enzyme and muscarinic receptors, acetylcholinesterase (**A**), M1 receptor (**B**), and M2 receptor (**C**) expression during experimental *T. cruzi* infection. Bars indicate mean ± SD. The differences are considered statistically significant when *p* < 0.05 and are demonstrated by different letters (A–C); the same letters represent no difference between groups (AB, BC and AC).

**Figure 4:**
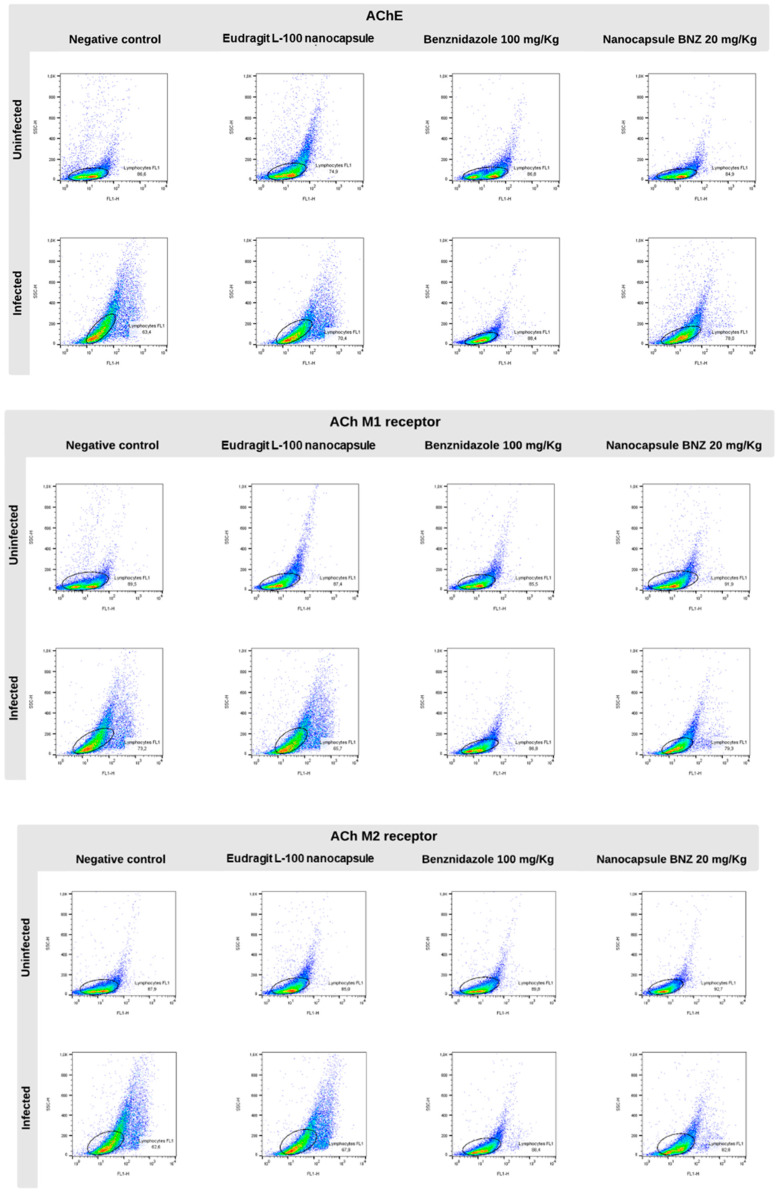
Representative lymphocyte cytometry was marked with cholinergic enzymes and muscarinic receptors during the experimental *T. cruzi* infection and plotted gates representation of AChE, AchM1, and AchM2 marker events. The circles represent the major expression gates of AChE, AchM1 and AchM2, while the color shown the marker fluorescence.

Moreover, flow cytometry was used to analyze the AChE expression of lymphocytes and the muscarinic receptors (M1 and M2) involved in immune response. The AChE expression (Figure 3A and Figure 4) was lower in the infected CT group than in the uninfected CT group, as can be observed in the representative histograms. However, there was a significant increase in AChE expression in the infected BNZ group compared to that in the infected CT group.

The muscarinic receptors M1 and M2 (Figure 3B,C and Figure 4) showed significant expression in the infected CT and EL-100 groups in relation to their uninfected groups. The infected BNZ group showed a more substantial receptor expression than the infected CT group. Moreover, the NBNZ’s M1 and M2 expression was higher compared to that of the uninfected group and the infected CT group.

**Figure 5:**
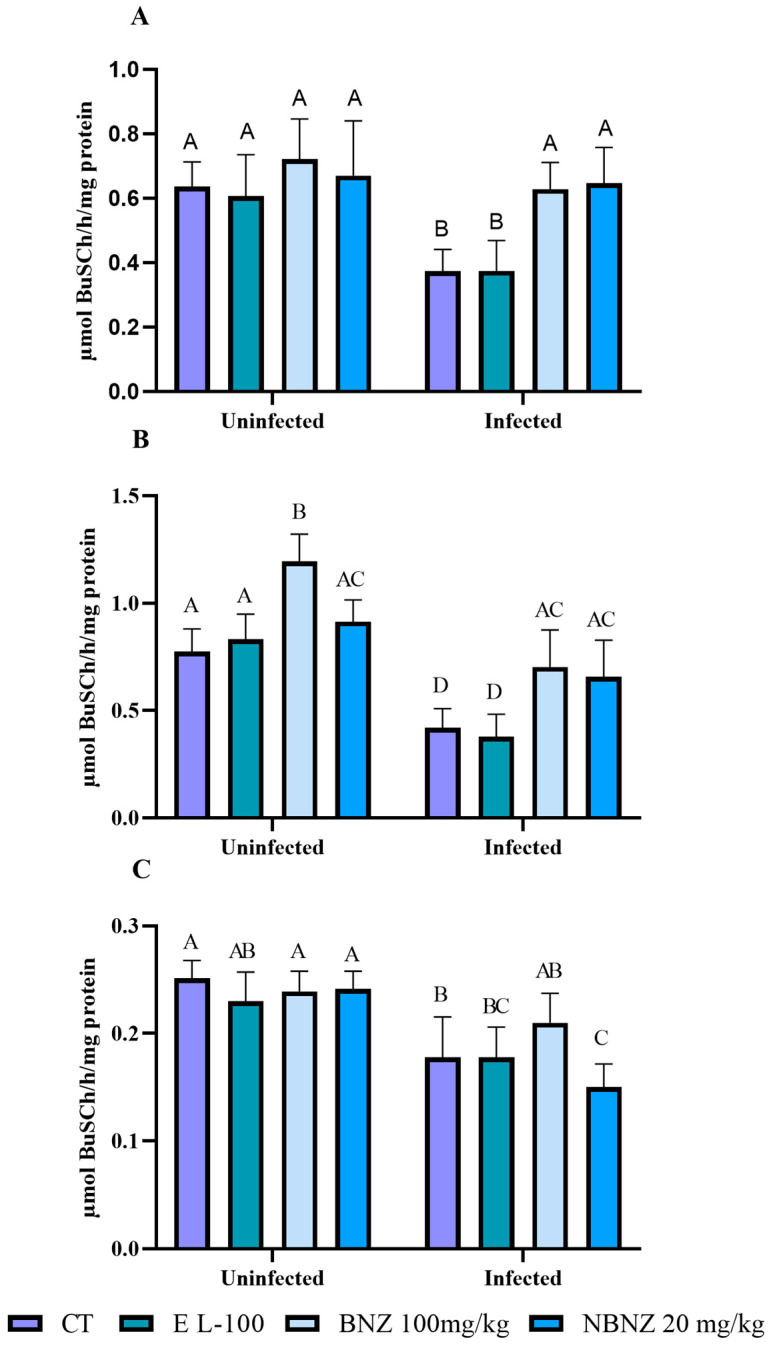
Butyrylcholinesterase activity during experimental acute *T. cruzi* infection in Swiss mice: plasma (**A**), liver (**B**), and cortex (**C**). Bars indicate mean ± SD. The differences are considered statistically significant when *p* < 0.05 and are demonstrated by different letters (A–D); the same letters represent no difference between groups (AB, BC, and AC).

Plasma BuChE activity (Figure 5A) was lower in the infected CT and EL-100 groups than in the uninfected CT and EL-100 groups. The infected BNZ and NBNZ groups demonstrate significant enzyme activity compared to the infected CT group, based on the basal enzyme levels.

The enzyme activity in the liver (Figure 5B) is lower in the infected CT, EL-100, and BNZ groups than in their corresponding uninfected groups. Moreover, there was no difference in enzyme activity between the infected NBNZ and the uninfected NBNZ group. Still, the infected BNZ group showed significant enzyme activity when compared to the infected CT group. In the cortex (Figure 5C), a lower BuChE activity was observed in the infected CT and NBNZ groups compared to their corresponding uninfected groups. In addition, the infected NBNZ group showed a lower BuChE activity than the infected CT group.

### 2.5. Oxidative Status on Acute T. cruzi Infection

**Figure 6:**
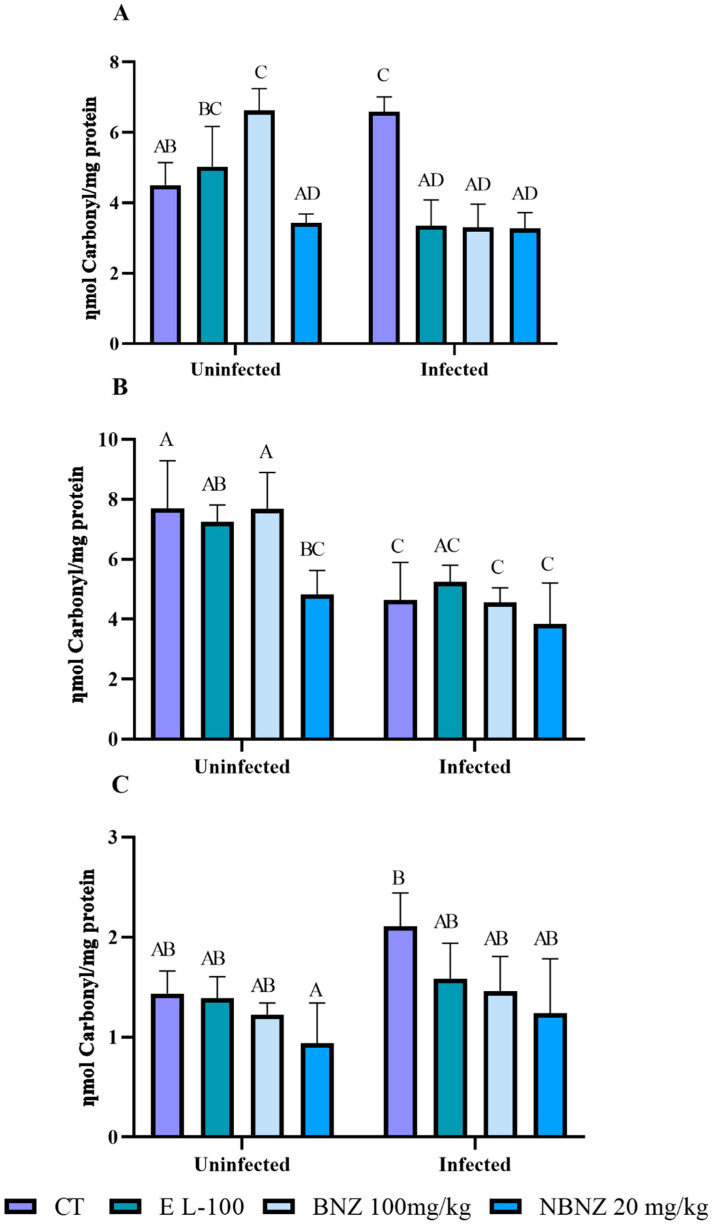
Evaluation of protein carbonylation during experimental acute *T. cruzi* infection of Swiss mice liver (**A**), kidney (**B**), and cortex (**C**). Bars indicate mean ± SD. The differences are considered statistically significant when *p* < 0.05 and are demonstrated by different letters (A–D); the same letters represent no difference between groups (AB, BC, AC, and AD).

Oxidative stress was evaluated in the liver, kidney, and cortex tissue using several oxidative parameters. Protein carbonylation in the liver (Figure 6A) was significant in the infected CT group compared to the uninfected CT group. However, protein carbonylation in the liver was lower in the infected EL-100 and BNZ groups than in their corresponding uninfected groups and the infected CT group. The infected NBNZ showed no difference in protein carbonylation in the liver compared to its uninfected group, but protein carbonylation was lower in the infected NBNZ than the infected CT group.

The renal protein carbonylation (Figure 6B) was lower in the infected than the uninfected groups. This decrease was significant in the infected CT and BNZ groups compared to their corresponding uninfected groups. No difference in renal protein carbonylation was observed between the uninfected and infected NBNZ groups. In the cortex (Figure 6C), the protein carbonylation concentration was higher in the infected CT group compared to the uninfected CT group; the other comparisons had no significant differences.

**Figure 7:**
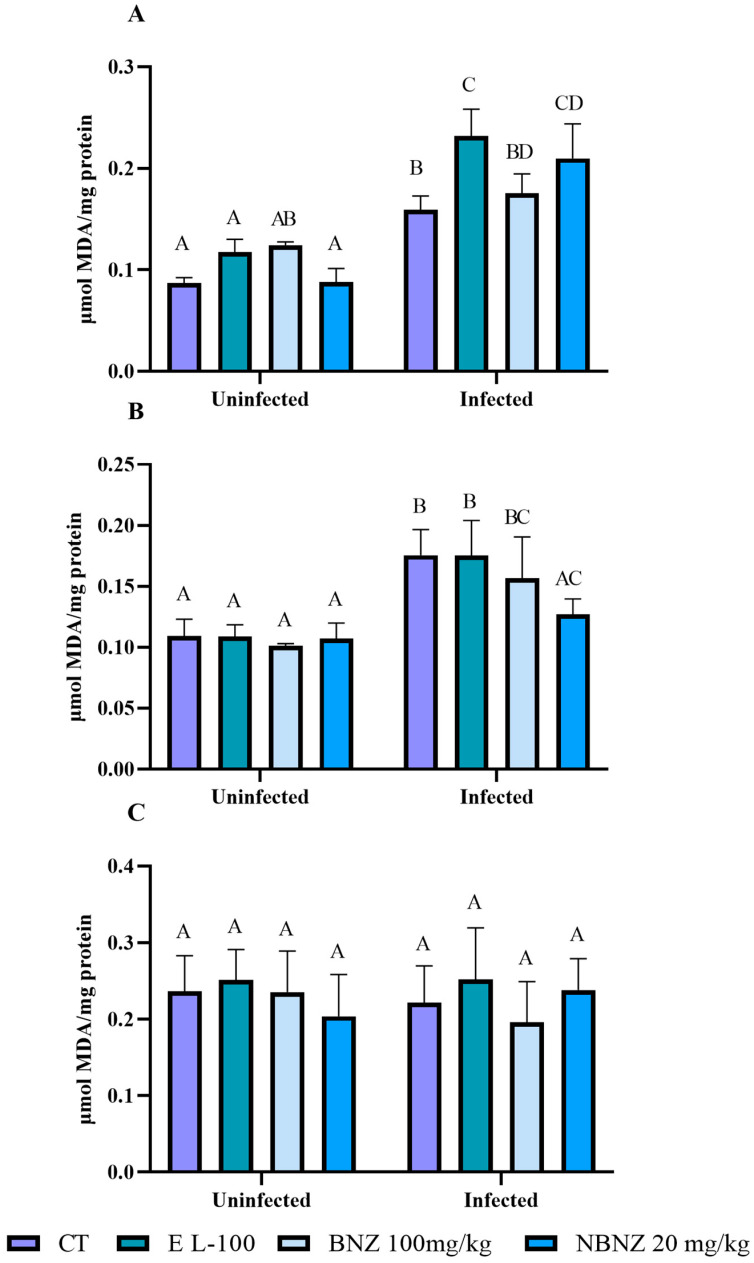
Evaluation of MDA levels during experimental acute *T. cruzi* infection of Swiss mice liver (**A**), kidney (**B**), and cortex (**C**). Bars indicate mean ± SD. The differences are considered statistically significant when *p* < 0.05 and are demonstrated by different letters (A–D); the same letters represent no difference between groups (AB, BC, AC, BD, and CD).

Malondialdehyde levels in the liver tissue (Figure 7A) were higher in all infected groups compared to all uninfected groups. The infected CT, EL-100, BNZ, and NBNZ groups showed higher MDA levels than the uninfected CT, EL-100, BNZ, and NBNZ groups. In addition, the infected EL-100 and NBNZ groups demonstrated higher TBARS levels compared to the infected CT group.

In the kidney (Figure 7B) analysis, a similar standard to the liver was observed, with more significant MDA levels in the infected CT, EL-100, and BNZ groups than in other groups. However, the infected NBNZ groups had no significant difference compared to the uninfected groups but showed lower MDA levels than the infected CT group. The evaluation of the cortex MDA levels (Figure 7C) showed no significant differences between all groups.

**Figure 8:**
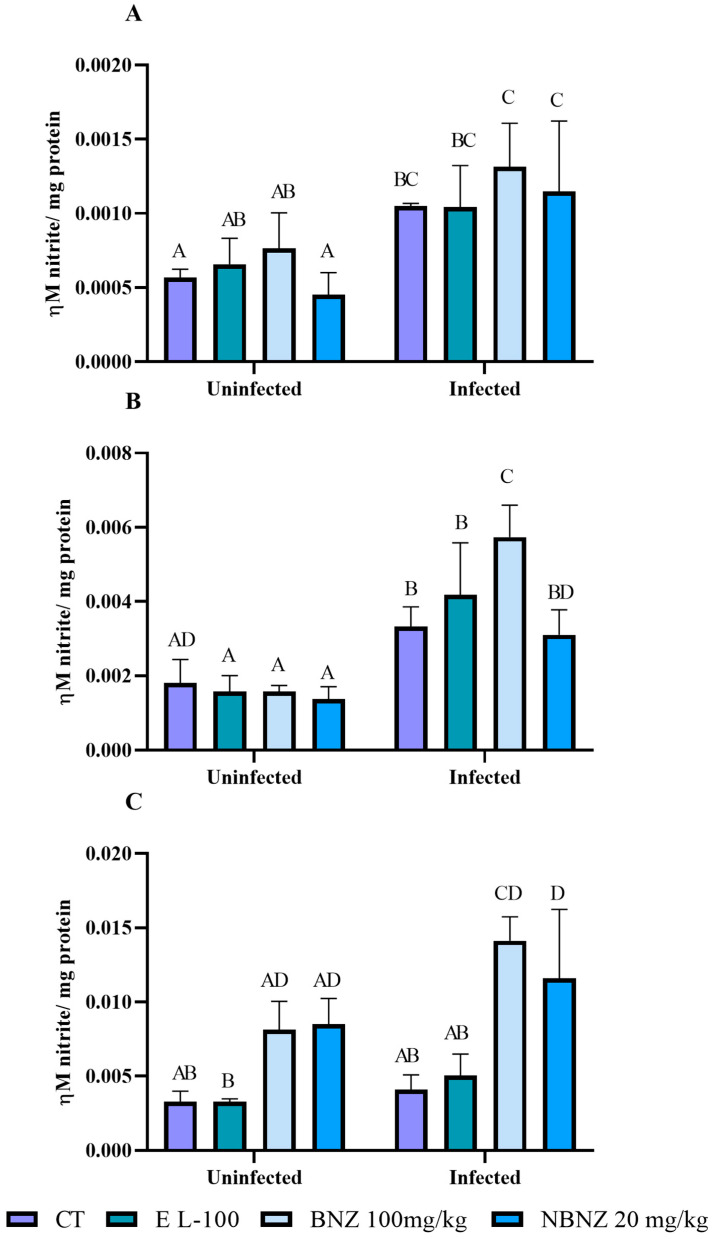
Evaluation of nitrate levels in Swiss mice liver (**A**), kidney (**B**), and cortex (**C**) during experimental acute *T. cruzi* infection. Bars indicate mean ± SD. The differences are considered statistically significant when *p* < 0.05 and are demonstrated by different letters (A–D); the same letters represent no difference between groups (AB, CD, BC, BD and AD).

Nitrite levels in the liver sample (Figure 8A) were higher in the infected CT, BNZ, and NBZ groups compared to their corresponding uninfected groups. In addition, the BNZ and NBNZ promote primary NOx levels in the infected groups compared to the infected CT groups. There were no differences between treatments within the uninfected groups and the infected groups. A similar result to the liver was observed for the NOx kidney (Figure 8B). The NOx levels in the infected groups (CT, EL-100, BNZ, and NBNZ) were higher compared to their respective uninfected groups. Moreover, the infected BNZ group showed primary NOx levels compared to other infected groups. The cortex NOx levels (Figure 8C) were more significant in the infected BNZ and NBNZ groups than in the infected CT and EL-100 groups. The same can be observed in the comparison between the uninfected groups.

**Figure 9:**
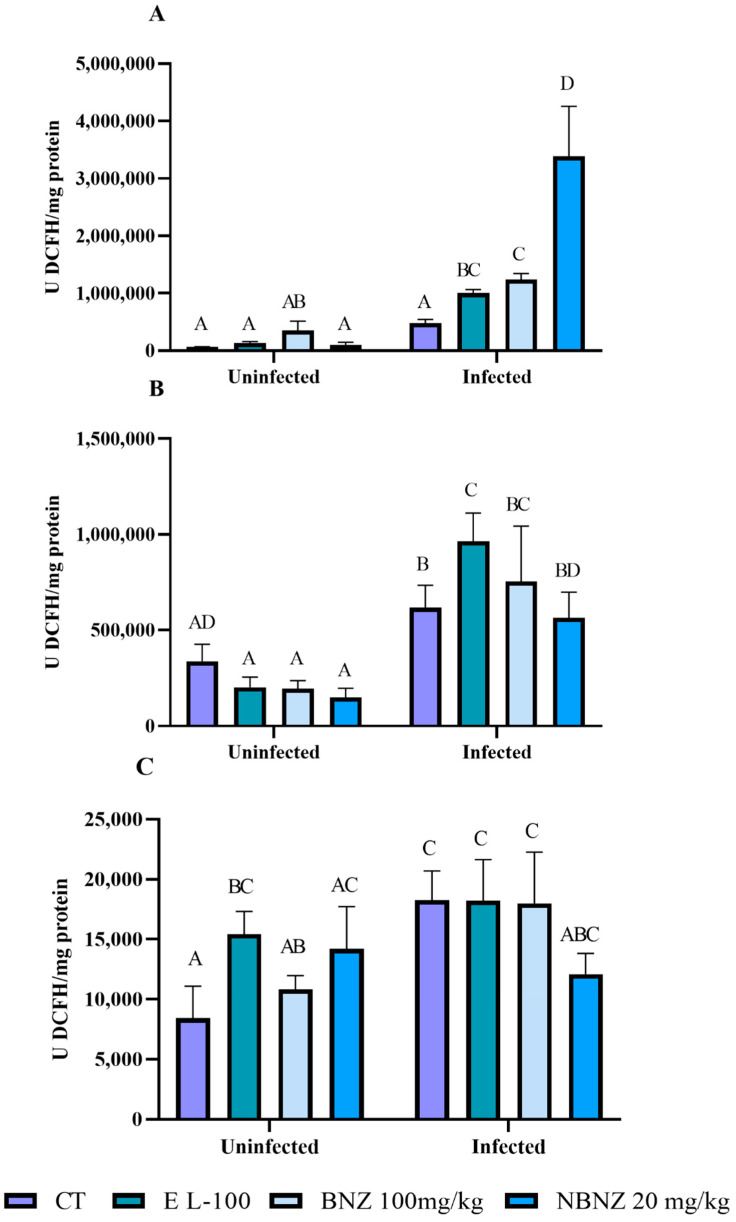
ROS levels during experimental acute *T. cruzi* infection of Swiss mice liver (**A**), kidney (**B**), and cortex (**C**). Bars indicate mean ± SD. The differences are considered statistically significant when *p* < 0.05 and are demonstrated by different letters (A–D); the same letters represent no difference between groups (AB, AC, ABC, AD, BC and BD).

The liver ROS levels (Figure 9A) were higher in the infected EL-100, BNZ, and NBNZ groups compared to their corresponding uninfected groups. Moreover, the infected EL-100, BNZ, and NBNZ groups demonstrated higher ROS levels than the infected CT group. In kidney evaluation (Figure 9B), more significant ROS levels were observed in all infected groups than in their respective uninfected groups. The cortex ROS levels (Figure 9C) were higher in the infected groups, and the difference in cortex ROS levels was significant between the infected and uninfected groups of CT and BNZ.

### 2.6. Evaluation of the Antioxidant Capacity of Free and Nanoencapsulated Benznidazole During T. cruzi Infection

**Figure 10:**
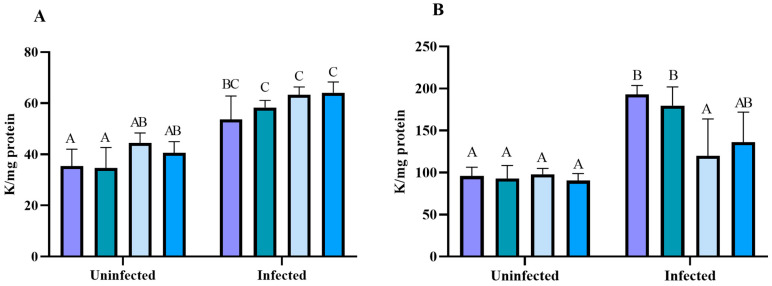
Catalase activity assay in experimental acute *T. cruzi* infection. Swiss mice liver (**A**), kidney (**B**) enzyme activity during Y strain infection. Bars indicate mean ± SD. The differences are considered statistically significant when *p* < 0.05 and are demonstrated by different letters (A–C); the same letters represent no difference between groups (AB and BC).

The antioxidant enzymes, catalase and superoxide dismutase, were evaluated. The liver CAT activity (Figure 10A) was more significant in the infected groups than in the uninfected groups of treatments. In the kidney (Figure 10B), a higher CAT enzyme activity was observed in the infected (CT and EL-100) groups in comparison to the uninfected groups. The mice treated with BNZ and NBNZ showed a lower CAT activity in the infected groups, with similar levels to those in the uninfected groups.

**Figure 11:**
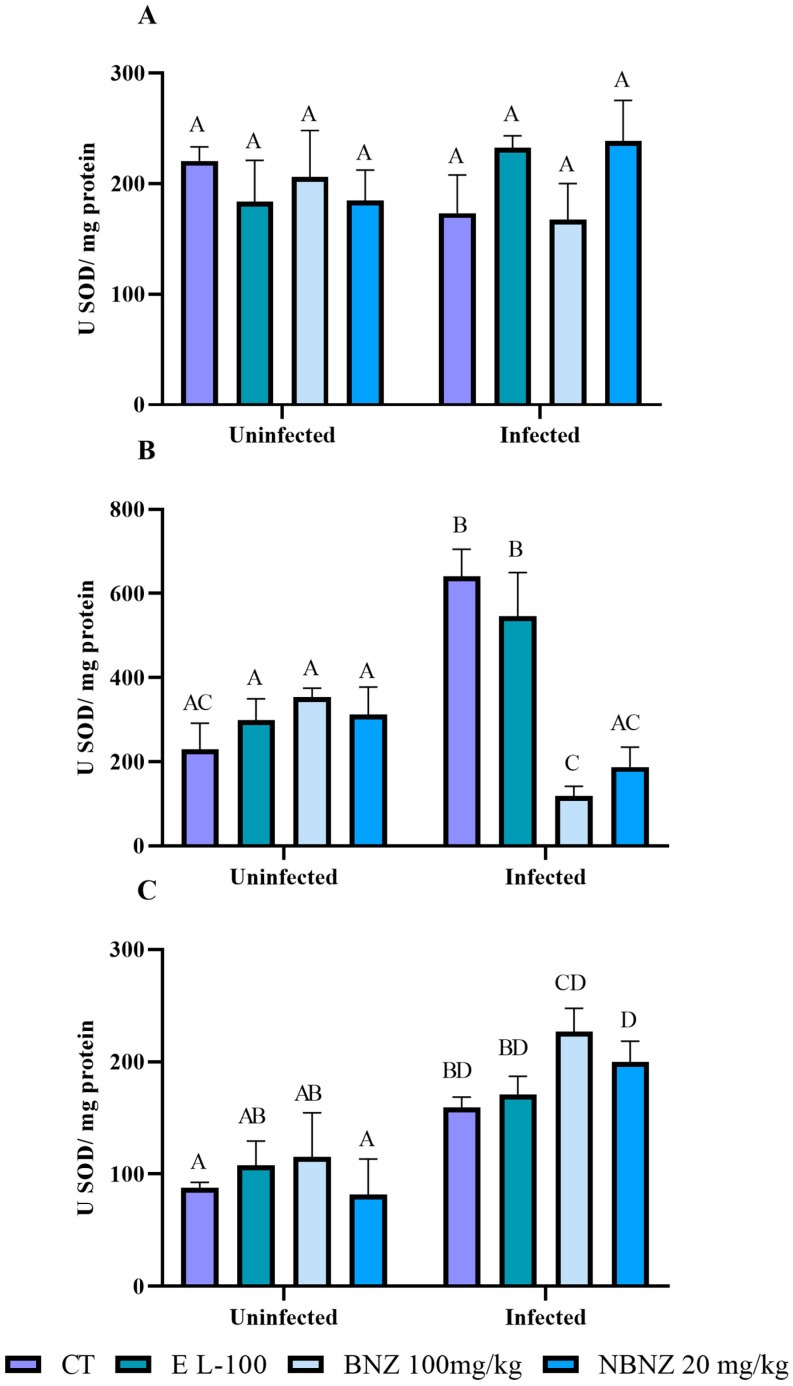
Superoxide dismutase activity assay in Swiss mice liver (**A**), kidney (**B**), and cortex (**C**) during Y strain infection. Bars indicate mean ± SD. The differences are considered statistically significant when *p* < 0.05 and are demonstrated by different letters (A–D); the same letters represent no difference between groups (AB, AC, BD and CD).

There was no significant difference in the SOD activity between groups in the liver tissue (Figure 11A). However, in the kidney tissue (Figure 11B), a more significant SOD activity was observed in the infected CT and EL-100 groups compared to their corresponding uninfected groups. On the other hand, the animals have a lower SOD activity in the infected BNZ and NBNZ groups than the infected CT and EL-100 groups. The cortex SOD activity (Figure 11C) was more significant in the infected groups (CT, BNZ, and NBNZ) than in their respective uninfected groups.

The glutathione-S transferase activity, TSH, and NPSH were evaluated in the liver, kidney, and cortex samples (Supplementary Material—Figure S1). The GTS activity in the liver (Figure S1A) was higher only in the infected BNZ group compared to the uninfected BNZ group; other groups presented no differences. The kidney (Figure S1B) and cortex (Figure S1C) enzyme activity had no differences in group comparison. The liver THS levels (Figure S2A) were more significant in the infected groups (CT, EL-100, and NBNZ) than in their corresponding uninfected groups. The THS levels of the kidney (Figure S2B) were higher in the infected BNZ group compared to its corresponding uninfected group. The cortex (Figure S2C) presents no differences between groups. The NPSH levels were evaluated in the liver, kidney, and cortex, and there were no differences between groups (Figure S3A–C). 

### 2.7. Histopathological Analysis

**Figure 12:**
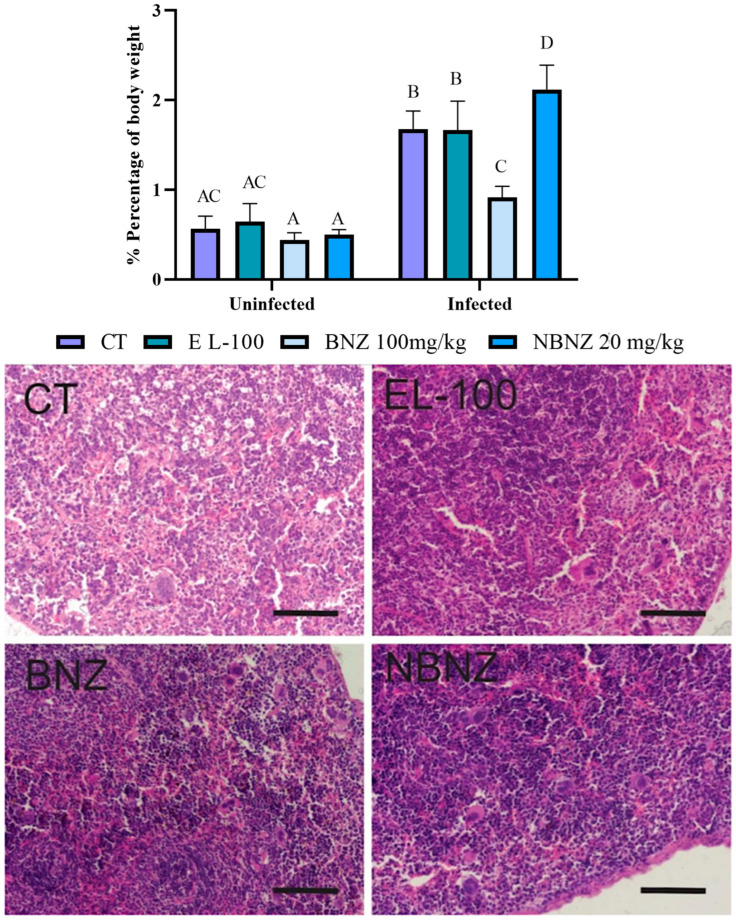
Spleen evaluation of mice experimentally infected by *T. cruzi*. Graphic demonstration of the spleen percentage of body weight (difference illustrated per different letter: A–D, the same letters represent no difference between groups (AC)). Photomicrography of the spleen tissue of mice experimentally infected by *T. cruzi*. CT: infected and untreated; EL-100: infected and treated with vehicle; BNZ: infected and treated with 100 mg/kg of BNZ; and NBNZ: infected and treated with 20 mg/kg of NBNZ. Stained with hematoxylin and eosin (H&E). Scale bar = 100 mm.

The analyze of splenomegaly (Figure 12) was assessed through a relation between spleen weight and body weight percentage. It was possible to see evidence of a more significant spleen weight in all infected groups than in uninfected groups. A lower percentage of body weight was observed in the infected BNZ group when compared to the infected CT group. The infected NBNZ group showed a more significant spleen weight than the infected CT group.

The histopathology of the spleen tissue (Figure 12) was evaluated through microscopy using an H&E stain. No parasitic forms were observed on the analyzed microscopic slides. Tissue samples of the CT group demonstrated preservation of tissue architecture without microscopic changes. The EL-100, BNZ, and NBNZ groups showed marked lymphocyte clonal expansion and the presence of activated macrophages scattered throughout the organ. In the BNZ and NBNZ groups, these cells were formed in a more abundant quantity.

**Figure 13:**
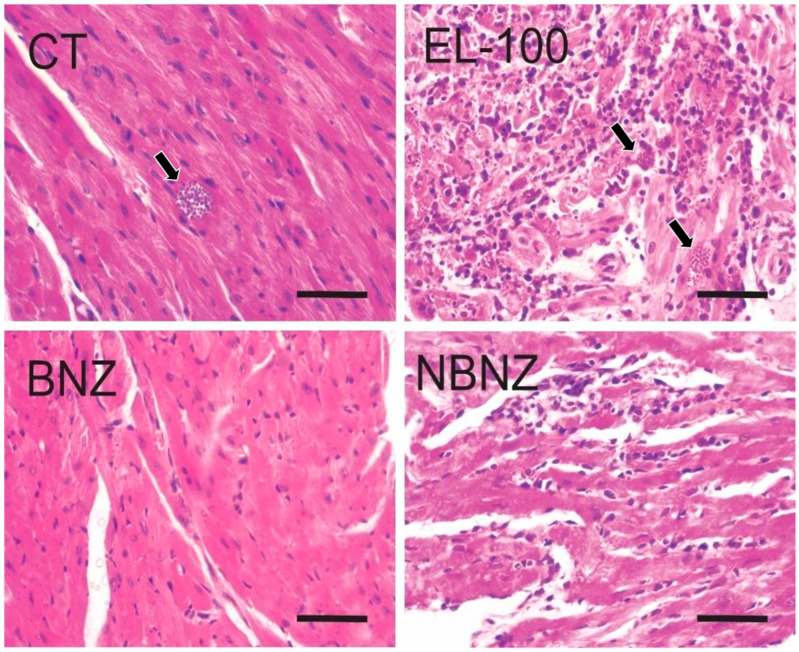
Histopathological images of the cardiac tissue of mice experimentally infected by *T. cruzi*. CT: infected and untreated; EL-100: infected and treated with vehicle; BNZ: infected and treated with 100 mg/kg of BNZ; and NBNZ: infected and treated with 20 mg/kg of NBNZ. Arrows show amastigote nests. Stained by H&E. Scale bar = 50 mm.

**Figure 14:**
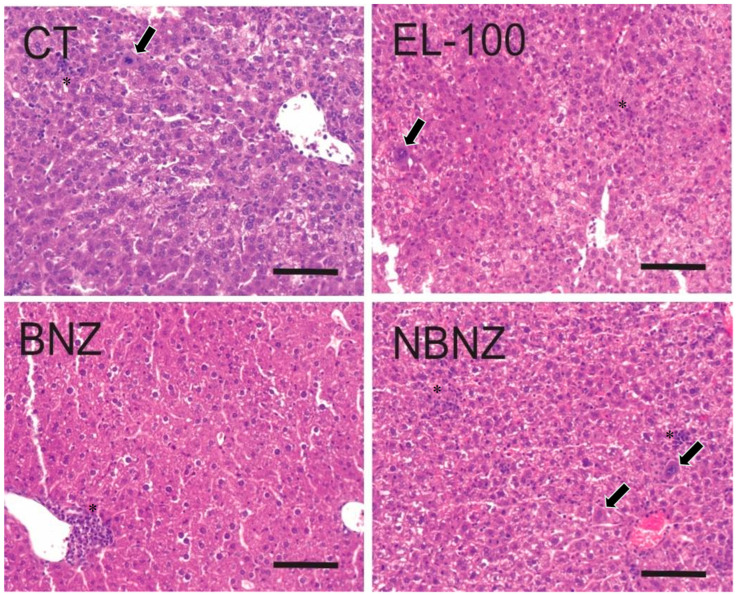
Histopathological images of the liver tissue of mice experimentally infected by *T. cruzi*. CT: infected and untreated; EL-100: infected and treated with vehicle; BNZ: infected and treated with 100 mg/kg of BNZ; and NBNZ: infected and treated with 20 mg/kg of NBNZ. * Inflammatory infiltrate; arrows show activated macrophages. Stained by H&E. Scale bar = 100 mm.

The cardiac histopathology (Figure 13) showed the presence of parasites in the CT, EL-100, and NBNZ groups. In the biopsies of the CT and EL-100 groups, extensive areas of inflammatory infiltrate, amastigote nests, and diffuse necrosis of cardiac tissue were observed. The samples from the other groups showed no alterations. In photomicrography of the liver tissue (Figure 14), it was possible to observe in the CT group that loss of integrity of hepatocyte cords, leukocyte clusters, presence of activated macrophages, and areas of necrosis were observed. In the samples of the EL-100 group, a vast leukocyte infiltration with activated macrophages, hepatic cords with structural loss, and vacuolated hepatocytes close to the centrilobular vein were observed.

In the liver of animals from the BNZ group, leukocyte infiltration was observed around the vessels but their structural integrity was maintained. In samples from the NBNZ group, abundant inflammatory infiltrate and activated macrophages were observed.

## 3. Discussion

Benznidazole is the first-choice drug for the treatment of CD in Brazil. However, this treatment promotes many adverse effects that can interrupt treatment [30,31,32]. In this context, the present study investigates the influence of conventional therapy and nanotechnology to suggest an enhanced and efficient therapeutic drug for CD. Hematological and biochemistry parameters, oxidant/antioxidant status, histology, and cholinergic molecular pathways were evaluated for this purpose.

*T. cruzi* is an obligate intracellular parasite; metacyclic trypomastigotes and bloodstream trypomastigotes are the infective forms [33]. In addition, the bloodstream trypomastigote can be identified to confirm the infection and accompany the parasitemia host. In parasitemia, the peak of infection occurred at 7 p.i. These results were also observed in other studies [20,34,35].

Nanotechnology has been used as a tool to diminish side effects. Here, nanostructures containing BNZ were produced in stable nanocapsules of Eudragit. In addition, drug nanocarriers against *T. cruzi* are being used to decrease the dose and increase the effects against the parasite, as reported by Sousa et al. [36], who showed that nanoformulations demonstrate low cytotoxicity when compared with conventional treatment. These findings are consistent with our study and data.

It is the knowledge that the toxicity of the BNZ is derived from the formation of nitroradical anions and reactive product generation when interacting with molecular DNA [37,38]. In this sense, we investigate whether the NBNZ could also diminish parasitemia through the qPCR technique. It was notorious for the presence of genomic DNA of *T. cruzi* in the cardiac and nervous tissues of animals treated with BNZ, causing a low efficacy of treatment in situ. These data are from a previous study and describe the BNZ efficacy to be about 76% in the acute phase [39,40]. The NBNZ showed a lower efficacy in the heart and a similar efficacy to the BNZ in the brain. Still, both tissues of NBNZ-treated infected animals have a genomic DNA of *T. cruzi*.

The analyzed hematological parameters have a substantial role in evaluating and accompanying CD and its treatment evolution. This analysis demonstrated an anemia state with reduced erythrocytes, hemoglobin, and hematocrit in the current research [41,42,43]. This condition was observed in our study in the infected groups. However, the BNZ prevented this hematological alteration, but the NBNZ does not present this action. Furthermore, the leukocytes present a reduction during the acute *T. cruzi* infection accompanied by granulocytopenia. However, the NBNZ was able to promote leukocytosis with granulocytosis and lymphocytosis. There was thrombocytopenia in the *T. cruzi* infection, which was reversed by the BNZ and NBNZ.

The physiopathology of anemia, leukopenia, and thrombocytopenia can be related to complex mechanisms involving an increased cell destruction or a decreased cell production. The infection may be associated with reduced cell precursors in the bone marrow, mainly erythroblast and megakaryoblast. The decrease in the numbers of erythrocytes, platelets, and leukocytes may result in a suppression in cell production in the bone marrow, which can be related to a side effect of the BNZ. On the other hand, anemia can be related to the interaction with parasite molecules in the blood and to an autoimmunity action of organisms that can occur during the infection [43,44,45].

Functional parameters such as AST and ALT are considered as hepatic damage markers but are related to cardiac tissue damage when altered. These parameters were evaluated to verify hepatotoxicity in CD and the effects of treatments. There was a pronounced increase in both markers in all infected experimental groups. Moreover, BNZ is a choice drug for acute CD treatment; this drug is metabolized in the liver and may promote hepatic toxicity, which can be related to an increase in AST and ALT activities in the initial weeks of treatment [32,46], as observed in this study.

In addition, creatinine and urea are used as renal injury markers. These parameters were increased in the infected groups. However, the treatments were able to control these alterations. The BNZ-infected animals present an increase in creatinine levels. Still, there were no tissue alterations in the histopathological analyses, which can be related to the BNZ metabolism that is partially renal. It has been related to renal injury in acute CD, but the intensity of the inflammatory process may be linked with the parasite’s infection load [46]. Some research studies have described that renal alterations and compromise are not associated with the parasite’s presence but with infiltrated inflammation as a disease complication. Cardiovascular dysfunction is one of the main complications in acute CD and can lead to a decreased renal blood flow, which promotes several alterations in tissue conditions [47,48].

The cholinergic system has been related to the immune response in parasitic infections [20,49]. This system plays a vital role in inflammatory and immune response modulation [20,50,51,52]. Here, we evaluated some cholinergic system components in lymphocytes isolated from the spleen. This organ is an essential target involved in immune responses. Furthermore, the AChE enzyme was assessed in total blood and cortex. The BuChE activity was analyzed in the plasma, liver, and cortex. Our data showed an increased AChE activity in lymphocytes, the blood, and the cortex in the acute *T. cruzi* infection. The NBNZ promoted a reduction in AChE in uninfected lymphocytes, but this treatment promoted an increased enzymatic activity in blood. AChE is responsible for hydrolyzing acetylcholine, which has an inflammatory response. Once the AChE is increased, it is possible to suggest that accelerated hydrolysis can reduce the ACh levels. ACh is a neurotransmitter but is too produced by mononuclear immune cells and may act by autocrine and paracrine forms for immune regulation, interacting with muscarinic and nicotinic AChR [51,52].

M1 and M2 mAChR expression was evaluated, and a decrease was demonstrated during acute *T. cruzi* infection. However, the infected animals that underwent the BNZ presented an increased expression of M1 and M2 mAChR. The AChE expression showed the same pattern as mAChR. The reduced expression may be linked to a control mechanism of the inflammatory process since the reduction in the AChE levels can attenuate the inflammation promoted by immune cells. This reduction is associated with AChE binding to M1 and M2 receptors. The M1 mAChR suppression may be related to a reduction in TNF-α, INF-γ, and IL-6; that is, these receptor types are involved in regulating pro-inflammatory response and modulating antibody class transition from IgM to IgG [51,52].

The M1 is a muscarinic receptor coupled to Gq/11; when stimulated, it mediates the phospholipase C activation that increases intracellular Ca^2+^. The M2 mAChR subtypes are coupled to Gi/o; under stimulation, they promote the inhibition of adenylyl cyclase, resulting in a cAMP synthesis reduction [50]. However, M2 needs to be explored more in anti-inflammatory cholinergic pathway studies, which require a clearly described role. However, this research showed an expression pattern very similar to M1 mAChR. Some researchers showed that M1/M5 KO mice demonstrate a suppression in AChE gene expression, suggesting that M1 and/or M5 mAChR may modulate AChE transcription, and it occurs in an independent form of α7 nAChRs. This indicates that an upregulation of immunological stimulation occurs by ACh synthesis and its degradation [51,52].

The AChE activity in the blood and brain was previously evaluated. It showed a reduced enzyme activity related to decreased parasitemia in infected and untreated animals [20]. The cholinergic modulation showed a different pattern in the blood and brain that can be associated with a second increase in parasitemia after day 9 p.i. This increase in parasitemia may promote a significant inflammatory response in these cells since the process and presentation of antigens are factors that may stimulate the pro-inflammatory modulation in these cells [51,52]. In addition, a reduced BuChE activity was observed in the infected groups. This enzyme also hydrolyzes ACh; AChE had a higher catalytic efficiency than BuChE in substrate hydrolysis [53]. This reduced activity may be a compensatory mechanism to regulate the inflammatory response in acute infection once ACh produces an anti-inflammatory response.

The redox status was measured in the present study. Carbonyl levels are associated with protein oxidation [54] and were elevated in this study. Fracasso et al. [55] also showed decreased carbonyl levels in the liver in the acute phase of CD, corroborating our data of the infected and treated groups. Furthermore, the infected and untreated groups evidence an opposite pattern. In addition, the TBARS levels indicate lipid peroxidation related to membrane integrity and a loss of membrane protein function [56]. The more significant TBARS levels in the liver and kidney in our findings were also described by Barbosa et al. [57] and Fracasso et al. [55].

NOx and ROS are molecules associated with the inactivation of trypomastigotes, mediated by the BNZ molecule. The BNZ exerts its trypanocidal effects after enzymatic activation by trypanosomal type I nitroreductases (NTRs), according to Patterson and Wylle [58]. Furthermore, *T. cruzi*-derived inflammation recruits immune cells and produces ROS/RNS [59,60]. Fracasso et al. [55] reported similar results to those shown here. Therefore, trypomastigotes forms are likely the targets of NOx, resulting in the killing of the parasite [61]. The increased ROS/RNS levels influence the production of other inflammatory cytokines that contribute to the exacerbation of inflammatory responses mediated by *T. cruzi* responses, as demonstrated in other studies [20].

Antioxidant enzymes also balance the redox status. Catalase, according to Baldissera et al. [62], is an enzyme that acts by degrading hydrogen peroxide. While the superoxide dismutase (SOD) enzyme acts by dismuting the superoxide radical into hydrogen peroxide [63,64]. Here, the data show an elevated CAT activity in infected animals, corroborating previous results [55,62]. The increase in the SOD activity in *T. cruzi*-infected animals may depend on the type of tissue involved in the activation of other O−2 dismutation pathways. The SOD activity could not reverse the ROS levels [65,66]. The levels of GST, TSH, and NPSH did not show statistical significance. Still, they may be associated with one compensatory mechanism, where the host uses other ways of controlling the parasitemia levels and acts to reduce the pro-inflammatory process induced by *T. cruzi*.

*T. cruzi* infects the host through mucosa invasion. The trypomastigote form of the parasite invades mainly macrophages; however, it can infect several cell types in diverse tissues, like the heart, smooth muscle in the digestive tract, esophagus, lungs, kidney, spleen, liver, brain, placenta, bone marrow, and others [67]. The brain, heart, liver, kidney, and spleen were histopathologically evaluated in this experiment. The parasite amastigote forms were found only in the cardiac tissue. Other tissue analyses, such as those of the liver and spleen, showed significant inflammatory infiltration and tissue damage. Thus, the BNZ, but not the NBNZ, prevents the cardiac tissue invasion by the parasite.

## 4. Materials and Methods

### 4.1. Benznidazole Nanocapsule Development

Benznidazole (N-Benzyl-2-nitro-1H-imidazole-1-acetamide) (97%) was acquired from Sigma Aldrich (São Paulo, Brazil), and the Eudragit L100 polymer was kindly donated by the Evonik company (São Paulo, Brazil).

To develop nanocapsules containing benznidazole (1 mg/mL), the preformed polymer interfacial deposition method [29] was used, with modifications. The organic phase contained the Eudragit L100 polymer (0.25 g), benznidazole (0.025 g), sorbitan monooleate (0.19 g), medium-chain triglycerides (413 μL), and ethanol (67 mL), and the aqueous phase contained polysorbate 80 (0.19 g) and ultrapure water (134 mL). Then, the formulation was submitted to evaporate the organic solvent under reduced pressure to the final volume (25 mL), with temperature control at 40 °C.

### 4.2. Experimental Design I

Fifty female Swiss mice were distributed in eight groups: CT—negative control (uninfected animals, n = 6); EL-100—EL-100 nanocapsule (uninfected animals, n = 6); BNZ—free benznidazole (100 mg/kg) (uninfected animals, n = 6); NBNZ—benznidazole nanocapsule (20 mg/kg) (uninfected animals, n = 6); infected CT—positive control (infected animals, n = 8); infected EL-100—EL-100 nanocapsule (infected animals, n = 6); infected BNZ—free benznidazole (100 mg/kg) (infected animals, n = 6); and infected NBNZH—benznidazole nanocapsule (20 mg/kg) (infected animals, n = 6). The infected groups were infected with *T. cruzi* (Y strain), with 1 × 10^4^ trypomastigote forms, by intraperitoneal injection. Six animals were distributed in each group, except the positive control group, with eight animals in the experimental group.

#### 4.2.1. Treatment

Protocol treatments were made using nanoencapsulated benznidazole (C_12_H_12_N_4_O_3_, Sigma Aldrich) (20 mg/kg) according to Dutra da Silva et al. [68] and benznidazole pills of 100 mg [55,68,69] (the standard dose of the literature). After confirming the infection, the treatment groups received their respective treatments by orally gavage every day over eight days, and the parasitemia was analyzed every two days.

#### 4.2.2. Animals and Infection

The animals were submitted to a week of acclimatation before the infection and treatment. During the experiment, animals were kept in light/dark cycles (12 h) with controlled temperature and humidity (25 °C and 70%, respectively) and water and food ad libtum. At day 11 post-infection, the mice were euthanized. Animals were anesthetized using an isoflurane chamber and were euthanized by cardiac function according to Ethics Committee Protocols. All animals’ manipulation was approved by the animal welfare ethics committee of the Federal University of Santa Maria (approval number: 2842070618). Blood was collected in tubes containing EDTA; samples were processed immediately after collection. The cortex, heart, liver, spleen, and kidney samples were collected and stored at −80 °C until analysis. A fragment of tissues was kept in a 10% formalin solution for histological analysis.

In summary, the treatment was administered by oral gavage to all experimental groups. The infected and uninfected BNZ groups received a benznidazole dose of 100 mg/kg for eight days. At the same time, the infected and uninfected NBNZ groups received the nanocapsule benznidazole dose of 20 mg/kg in the same period. Furthermore, the EL-100 groups received the nanocapsule vehicle at the same volume as the NBNZ group every day over eight days. The infected and uninfected animal groups received the treatment. The gavage with filtered water was performed on animals of the positive and negative control group each day for eight days

### 4.3. Monitoring of Parasitemia Evolution

Parasitemia was followed by quantifying trypomastigotes in total blood, according to Brener’s methodology [70]. A blood sample was collected from the caudal vein of infected mice every two days.

For infection confirmation, Real-time Polymerase Chain Reaction (qPCR) was performed in the cardiac and cortex samples from three mice of each group at day 12 p.i. DNA was extracted from the samples using the method described by Lachaud et al. [71]. The detection of *T. cruzi* was performed by the amplification of a 188 bp nuclear fragment of the parasite DNA using two specific primers: TCZ-1 (5′-CGA GCT CTT GCC CAC ACG GGT GCT-3′) and TCZ-2 (5′-CCT CCA AGC AGC GGA TAG TTC AGG-3′). The RT-PCR was performed using KAPA SYBR Fast qPCR Master Mix (2X) (Kapa Biosystems, Ballardvale, Wilmington, MA, USA) with 10 ng of genomic DNA (gDNA). The samples were amplified with a Rotor-Gene Q (Quiagen, Netherlands, Germany) thermal cycler.

### 4.4. Hematological Parameter Evaluation

Hematological analysis was performed in an electronic counter (BC-2800 Vet—Auto Hematology Analyzer, Mindray^®^, Shenzhen Mindray Bio-Medical Electronics Co., Ltd., Shenzhen, China) for samples packed in tubes containing EDTA, and the determined parameters are as follows: total erythrocyte concentration (/µL), hemoglobin concentration (g/dL), platelet concentration (/µL), and total leukocyte concentration (/µL). The leukocyte differential was performed under immersion microscopy, at 1000× magnification, from blood smears made on a glass slide and stained with rapid Panoptic (Diff-Quick^®,^ Digilab, Piracicaba, São Paulo, Brazil). Total plasma protein (PPT) was verified by refractometry using the plasma portion of the microhematocrit capillary.

### 4.5. Biochemical Parameters

Biochemical analyses (AST, ALT, creatinine, and urea) were performed using a commercial kit (Bioclin^®^, Química Básica Ltda., Belo Horizonte, Minas Gerais, Brazil) for the serum from the animals according to the manufacturer’s instructions. The samples were processed in an automatic biochemical analyzer (Mindray BS-120^®^, Mindray Global, Shenzhen, China).

### 4.6. Mononuclear-Rich Lymphocyte Isolation from Spleen

The isolation of mononuclear-rich lymphocytes from the spleen was performed following Doleski et al. [72]. The organs were kindly homogenized in cold PBS-EDTA and centrifuged at 1500 rpm for 10 min. One wash was performed, suspending the pellet in PBS-EDTA. Ficoll-Hypaque was used to form the lymphocyte-rich mononuclear band, and after centrifugation, the band was collected. This isolate was submitted to the washing process, and the resultant pellet was washed and kept in ice.

### 4.7. Cholinergic System Evaluation

#### 4.7.1. AChE Assay in Lymphocytes

After the isolation of the lymphocytes, the AChE activity was determined according to the method described by Ellman et al. [73] and modified by Fitzgerald and Costa [74]. Briefly, the proteins of all samples were adjusted to 0.1–0.2 mg/mL. The amount of 0.2 mL of intact cells was added to a solution containing 1.0 mM acetylthiocholine, 0.1 mM of DTNB, and 0.1 M phosphate buffer (pH 8.0). Immediately before and after incubation for 30 min at 27 °C, the absorbance was read on a spectrophotometer at 412 nm. The results are expressed as μmol ACh/h/mg of protein.

#### 4.7.2. AChE Assay in Whole Blood

Whole blood AChE activity was determined by the method modified by Worek et al. [75]. Samples were hemolyzed with phosphate buffer, pH 7.4, containing Triton X-100, and stored at −30 °C for one week. The specific activity of whole blood AChE was calculated from the quotient between the AChE activity and hemoglobin content, and the results are expressed as mU ACh/μmol Hb.

#### 4.7.3. Cerebral AChE Enzymatic Assay

The AChE enzymatic assay was determined by modifying the spectrophotometric method of Rocha et al. [76]. The reaction mixture (300 µL final volume) contained 100 mM K^+-^phosphate buffer, pH 7.5, and 1 mM 5,5′-dithiobisnitrobenzoic acid (DTNB). The method is based on forming the yellow anion, 5,5′-dithio-bis-acid-nitrobenzoic, measured by absorbance at 412 nm during 2 min incubation at 25 °C. The enzyme (40–50 μg of protein) was pre-incubated for 2 min. The reaction was initiated by adding 0.8 mM acetylthiocholine iodide (AcSCh). All samples were run in duplicate or triplicate, and the enzyme activity was expressed in μmol AcSCh/h/mg of protein.

#### 4.7.4. BuChE

The plasma, liver and cortex tissue BuChE (EC 3.1.1.8; BUChE) activity was determined as described by Ellman et al. [73]. The BuChE activity was assayed in a medium containing sodium phosphate buffer 0.1 mM, pH 7.4, DTNB 0.30 mM, and 15 μL of serum. After 3 min of pre-incubation at 37 °C, the reaction was started with 1 mM of butyrylthiocoline (BuSCh), and the reading was performed for 2 min at intervals of 20 s in a spectrophotometer at 412 nm. The specific activity was expressed in μmol BuSCh/h/mg protein.

### 4.8. Lymphocyte Cytometry

The lymphocytes were fixed for 20 min in ice-cold 1% PFA in PBS, washed with PBS supplemented with 2% FBS, and incubated for 30 min with primary antibodies against AChE (1:200 Santa Cruz, CA, USA), AchM1 (1:200, Santa Cruz, CA, USA), and AChM2 (1:2000, Santa Cruz) receptors. The cells were incubated with Alexa Fluor 488- or 647-conjugated secondary antibodies (1:500) (Life Technologies, Carlsbad, CA, USA) and analyzed by flow cytometry (BD FACS Calibur; BD Biosciences, La Jolla, CA, USA). Thirty thousand events were acquired per sample. Data were analyzed using the FlowJo V10 software (FlowJo, BD Biosciences) and plotted in the histogram format.

### 4.9. Oxidative Parameters

The carbonylation of serum proteins was determined using a modified method [77]. Carbonyl content was calculated using 22 × 103 mmol/cm as the molar extinction coefficient, and the results were expressed as ηmoles of carbonyl groups per milligram protein.

Lipid peroxidation was measured as TBARS and expressed as the malondialdehyde (MDA) content. MDA, an end-product of fatty acid peroxidation, reacts with TBA to form a colored complex. The TBARS was analyzed in serum according to [78]. The results were expressed as ηmoles of malondialdehyde/mg of protein.

Nitric oxide levels were measured indirectly by evaluating nitrite/nitrate (NO_x_) content [79]. An aliquot (200 μL) was homogenized in 200 mM of Zn_2_SO_4_ and acetonitrile (96%, HPLC grade) for NO_x_ determination. The results were expressed as levels of NO/mg protein.

Reactive species were measured using 2′-7′-dichlorofluorescein (DCFH) fluorescence levels as an index of peroxide production by cellular components, according to Halliwell and Gutteridge [80]. Liver protein (0.8 μg) was added to a medium containing Tris–HCl buffer (10 mM; pH 7.4) and DCFH-DA (1 mM). The mixture medium was incubated in the dark for 1 h until the start of the fluorescence measurement procedure (excitation at 488 nm and emission at 525 nm, and both slit widths were 1.5 nm). The results were expressed as U DCF/mg protein.

### 4.10. Antioxidant Determination

The CAT activity in the tissue liver was determined by the decomposition of H_2_O_2_ at 240 ηm, according to the method described by Nelson and Kiesow [81] and modified by Aebi [82]. The results were expressed as ηmol CAT/mg protein. The SOD activity was measured according to the method of Misra and Fridovich [83]. The results are expressed as U SOD/mg protein. The GST activity was assayed spectrophotometrically at 340 ηm using the method of Habig et al. [84]. The activity was expressed as ηmol/h/mg of protein.

Thiols were measured spectrophotometrically using the method [85]. An aliquot of 100 μL for the liver in a final volume of 900 μL solution was used for the reaction. The reaction product was measured at 412 nm after the addition of 10 mM 5-5-dithio-bis (2-nitrobenzoic acid) (DTNB) (0.05 mL). A standard curve using cysteine was added to calculate the content of thiol groups in samples and was expressed as μmol TSH/mg protein.

For NPSH determination after protein precipitation, the resulting solution was centrifuged at 10,000× *g* for 5 min at 4 °C, and the free SH groups were determined in the supernatants. The reaction mixture consisting of 50 μL of sample, 450 μL of phosphate buffer, and 1.5 mL of 0.1 mM of 5′,5′-dithiobis 2-nitro benzoic acid was incubated for 10 min at 37 °C. The absorbance was measured at 412 nm using a SpectraMax plate reader (Molecular Devices, San Jose, CA, USA). The NPSH levels were expressed as μmol NPSH/mL.

### 4.11. Protein Determination

Protein content was determined using the Coomassie blue method, according to Bradford [86], with bovine sera albumin as the standard. The protein supernatant of the tissue was maintained at 0.6–0.8 mg/mL.

### 4.12. Histopathology Assay

The heart and liver fragments were collected and fixed in a 10% formalin buffer embedded in paraffin. Then, the microscope slides of 6 μm thick sections were made, stained with H&E, and analyzed under an optical microscope (Zeiss Axioscope A1, Oberkochen, Germany). The slides were scored for inflammation and defined by the degree of infiltration. The injuries were classified as mild, moderate, or severe.

### 4.13. Statistical Analysis

First, the data were subjected to normality testing (Shapiro–Wilk). All variables had a normal distribution, and parametric testing was performed. All statistical analyses were assessed using a two-way analysis of variance followed by Tukey’s test as a post-test using the Graph Pad Prism (Version 6.0) software. The results were expressed as mean ± standard error of the mean. The results were considered statistically significant when *p* < 0.05.

## 5. Conclusions

In summary, the present study evaluated the influence of the cholinergic signaling pathways on the immunomodulation and redox status during an acute CD *T. cruzi* infection and of benznidazole treatment on ACh hydrolysis, leading to a pro-inflammatory response. Nevertheless, the free BNZ was not 100% effective even with the highest dose in this treatment period. Still, it has continuously been a better option for treatment and can be associated with antioxidant components that can be evaluated in future research to protect the host organism. In addition, nanotechnology needs to be continuously tested for this disease because it represents an opportunity to improve treatment efficacy and quality of life of infected people.

## Figures and Tables

**Table 1:** Mean and SD of hematological parameters in *T. cruzi* infection.

Treatments		Erythrocytes (×10⁶/µL)	Hemoglobin (g/dL)	Hematocrit (%)	Platelets (/µL)	Leukocytes (/µL)	Granulocytes (/µL)	Lymphocytes (/µL)	Monocytes (/µL)
CT	UN	8.17 ^A^ ± 0.26	13.22 ^A^ ± 0.44	39.40 ^A^ ± 1.41	798666.67 ^A^ ± 207258.93	1833.33 ^AB^ ± 1427.81	426.33 ^AB^ ± 239.53	1351.67 ^AB^ ± 1090.71	55.33 ^A^ ± 104.50
	IN	5.17 ^B^ ± 0.17	8.16 ^B^ ± 0.26	24.40 ^B^ ± 0.69	392200.00 ^B^ ± 144771.19	1240.00 ^AB^ ± 536.65	163.20 ^B^ ± 81.15	1026.00 ^AB^ ± 494.17	50.80 ^A^ ± 43.47
EL-100	UN	8.00 ^A^ ± 0.54	12.95 ^A^ ± 0.76	38.48 ^A^ ± 2.48	791000.00 ^A^ ± 229249.50	2900.00 ^AB^ ± 1739.73	1017.50 ^AC^ ± 708.64	1706.00 ^AB^ ± 1056.41	176.50 ^A^ ± 161.68
	IN	5.38 ^B^ ± 0.74	8.375 ^B^ ± 1.34	25.02 ^B^ ± 3.26	425500.00 ^B^ ± 176722.94	3575.00 ^B^ ± 464.57	425.25 ^B^ ± 518.04	2882.50 ^B^ ± 666.54	267.25 ^A^ ± 214.43
BNZ (100 mg/kg)	UN	7.77 ^A^ ± 0.48	12.40 ^A^ ± 0.91	37.04 ^A^ ± 2.55	748000.00 ^AB^ ± 68025.73	2220.00 ^AB^ ± 1465.26	491.60 ^AB^ ± 201.37	1678.20 ^AB^ ± 1236.35	50.20 ^A^ ± 50.88
	IN	7.72 ^A^ ± 0.58	12.68 ^A^ ± 0.97	37.57 ^A^ ± 2.76	835833.33 ^A^ ± 116214.31	3116.67 ^AB^ ± 941.09	424.67 ^B^ ± 130.03	2639.17 ^AB^ ± 929.64	52.83 ^A^ ± 31.56
NBNZ (20 mg/kg)	UN	7.76 ^A^ ± 0.43	12.80 ^A^ ± 1.09	37.62 ^A^ ± 2.89	913500.00 ^A^ ± 141778.34	1216.67 ^A^ ± 552.87	306.67 ^B^ ± 112.91	859.00 ^A^ ± 413.83	51.00 ^A^ ± 37.79
	IN	6.01 ^B^ ± 0.60	9.45 ^B^ ± 1.01	28.92 ^B^ ± 2.80	780666.67 ^A^ ± 101653.66	5916.67 ^C^ ± 994.81	832.50 ^C^ ± 457.93	4902.50 ^C^ ± 957.21	181.67 ^A^ ± 112.77

The data are shown as mean ± SD. Statistical differences are considered when *p* < 0.05 and are represented by different letters, superscript (^A–C^).

**Table 2:** Mean and SD of liver and kidney function markers in serum.

Treatments		AST	ALT	Creatinine	Urea
CT	UN	126.50 ^A^ ± 9.81	31.00 ^A^ ± 1.15	0.43 ^A^ ± 0.049	52.00 ^A^ ± 3.46
	IN	448.25 ^B^ ± 5.5	174.5 ^C^ ± 5	0.625 ^BC^ ± 0.05	123.5 ^C^ ± 3.31
EL-100	UN	147.75 ^A^ ± 1.5	32.25 ^A^ ± 2.5	0.43 ^A^ ± 0.049	43.75 ^B^ ± 1.5
	IN	2764.25 ^C^ ± 10.5	241 ^D^ ± 4.76	0.53 ^AB^ ± 0.05	87.25 ^D^ ± 1.5
BNZ (100 mg/kg)	UN	132.33 ^A^ ± 15.37	24.25 ^B^ ± 3.40	0.56 ^A^ ± 0.12	43.75 ^B^ ± 2.5
	IN	144.5 ^A^ ± 3	37.75 ^E^ ± 1.5	0.68 ^C^ ± 0.05	45.25 ^B^ ± 2.87
NBNZ (20 mg/kg)	UN	99.00 ^A^ ± 6.92	23.50 ^B^ ± 5.19	0.45 ^A^ ± 0.05	36.50 ^B^ ± 0.57
	IN	807 ^D^ ± 34.43	61 ^F^ ± 1.63	0.53 ^AB^ ± 0.05	27.00 ^E^ ± 0.81

The data are shown as mean ± SD. The significant differences between groups are demonstrated through different letters, superscript (^A–F^). The differences are considered statistically significant when *p* < 0.05.

## Data Availability

All authors approved the manuscript for publication. This manuscript article includes all the data generated or analyzed during this study.

## Supplementary Materials

The following supporting information can be downloaded at https://www.mdpi.com/article/10.3390/ph17101397/s1, Figure S1. Glutathione transferase activity assay in experimental acute *T. cruzi* infection. Swiss mice liver (A), kidney (B), and cortex (C) enzyme activity during Y strain infection. Bars indicate mean ± SD. The differences are statistically significant when *p* < 0.05, demonstrated by different letters; the same letters represent no difference between groups. Figure S2. TSH levels in experimental acute *T. cruzi* infection. Swiss mice liver (A), kidney (B), and cortex (C) parameter during Y strain infection. Bars indicate mean ± SD. The differences are statistically significant when *p* < 0.05, demonstrated by different letters; the same letters represent no difference between groups; Figure S3. NPSH levels in experimental acute *T. cruzi* infection. Swiss mice liver (A), kidney (B), and cortex (C) parameter during Y strain infection. Bars indicate mean ± SD. The differences are statistically significant when *p* < 0.05, demonstrated by different letters; the same letters represent no difference between groups.
