# Supplementary material for: Effects of Free and Nanoencapsulated Benznidazole in Acute Trypanosoma cruzi Infection: Role of Cholinergic Pathway and Redox Status
Source: Pharmaceuticals (Basel). 2024 Oct 19;17(10):1397. doi: 10.3390/ph17101397 (PMC11510717; doi:10.3390/ph17101397)

Figure S1: Glutathione S-transferase activity assay in experimental acute *Trypanosoma cruzi* infection. Swiss mice liver (A), kidney (B) and cortex (C) enzyme activity during Y strain infection. Bars indicates mean ± SD. The differences are considered statistically significant when $p<0.05$ and these are demonstrated by different letters, same letters represent no difference between groups.

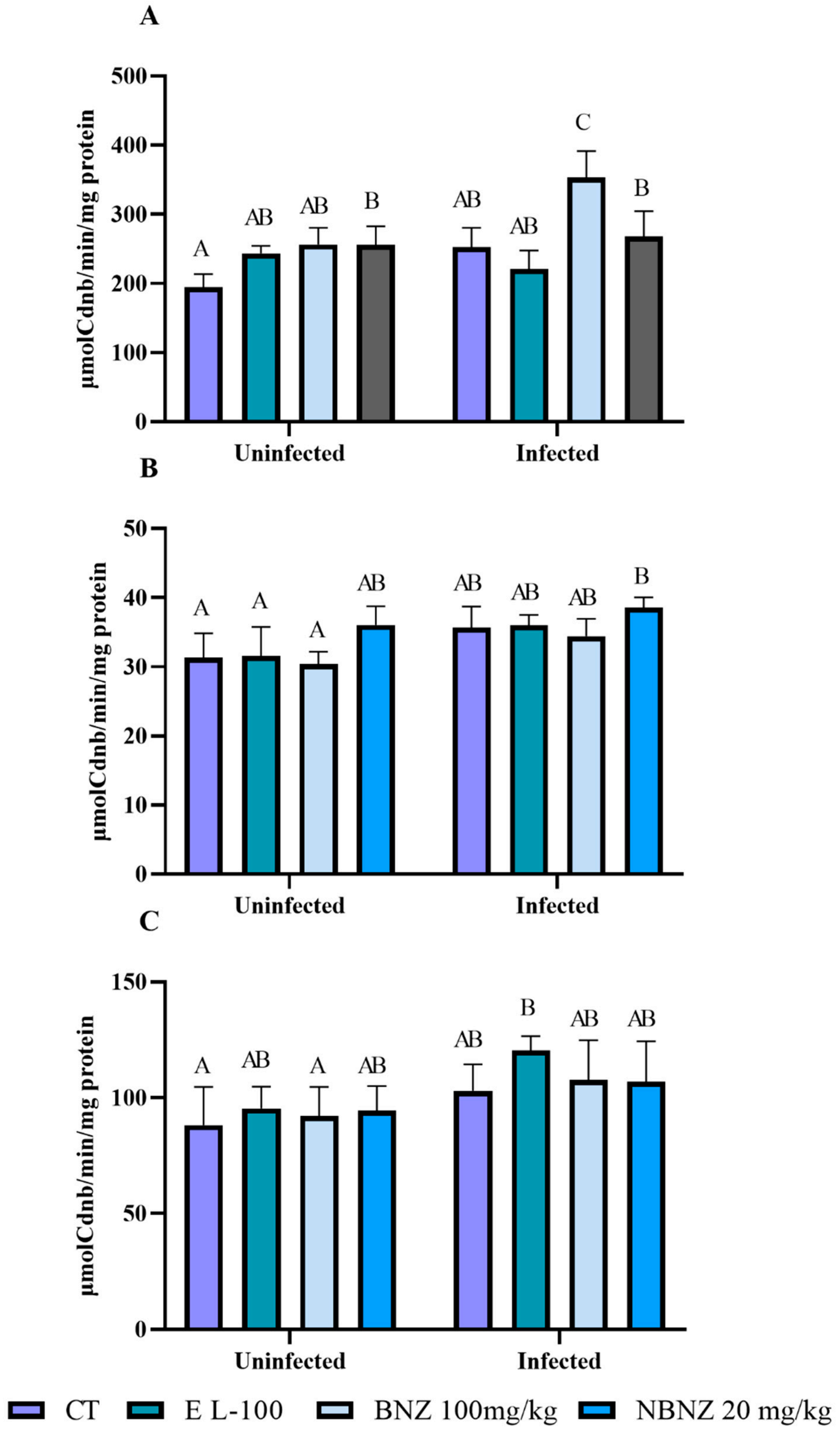

Figure S2: TSH levels in experimental acute *T. cruzi* infection. Swiss mice liver (A), kidney (B) and cortex (C) parameter during Y strain infection. Bars indicates mean ± SD. The differences are considered statistically significant when p<0.05 and these are demonstrated by different letters, same letters represent no difference between groups.

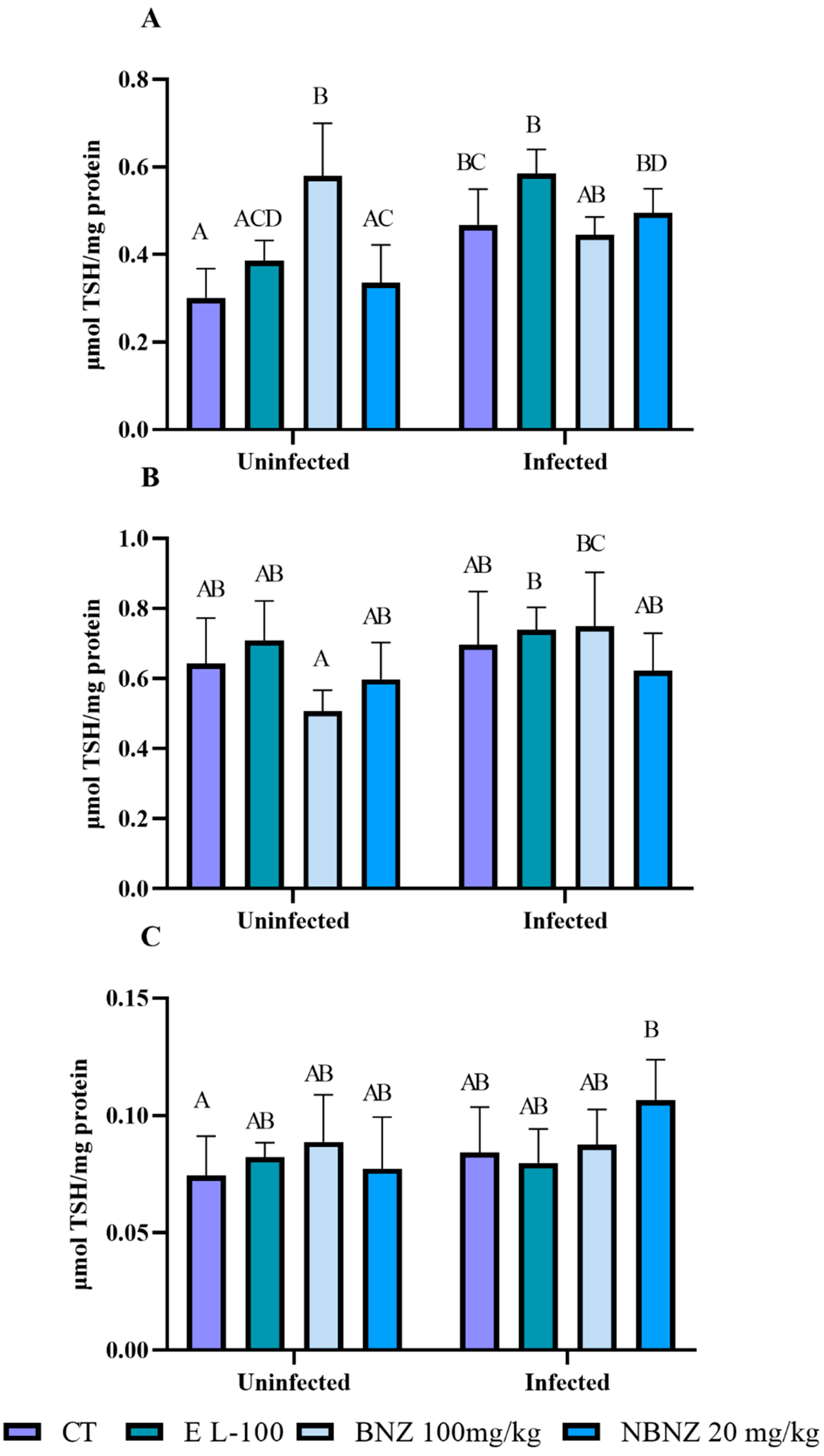

Figure S3: NPSH levels in experimental acute *T. cruzi* infection. Swiss mice liver (A), kidney (B) and cortex (C) parameter during Y strain infection. Bars indicates mean ± SD. The differences are considered statistically significant when $p<0.05$ and these are demonstrated by different letters, same letters represent no difference between groups.

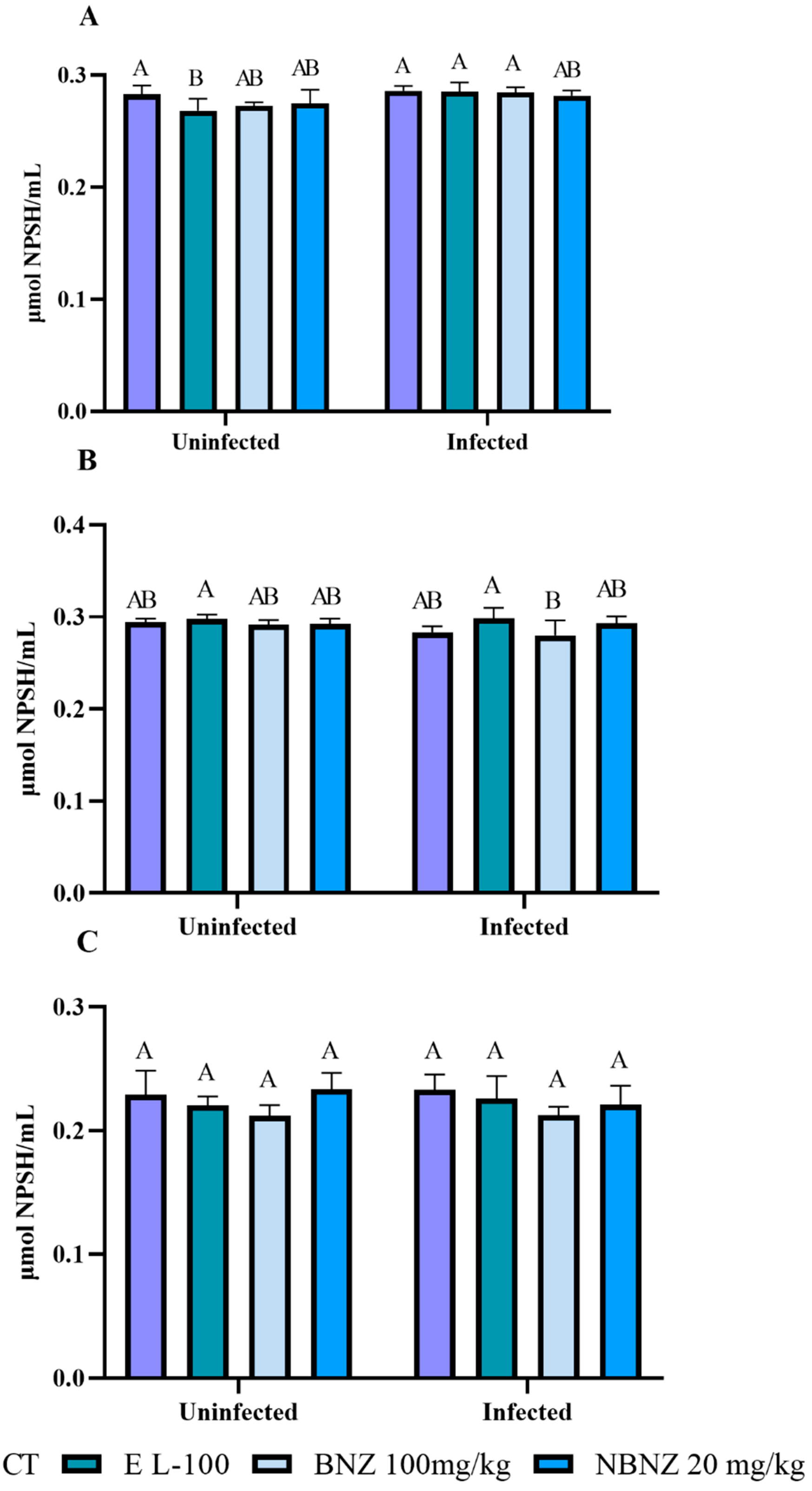

Supplement: Supplementary file 1 [file pharmaceuticals-17-01397-s001.zip › pharmaceuticals-3236646-supplementary.pdf]
